# SARS-CoV-2 outbreak: role of viral proteins and genomic diversity in virus infection and COVID-19 progression

**DOI:** 10.1186/s12985-024-02342-w

**Published:** 2024-03-27

**Authors:** Hosni A. M. Hussein, Ali A. Thabet, Ahmed A. Wardany, Ahmed M. El-Adly, Mohamed Ali, Mohamed E. A. Hassan, Mohamed A. B. Abdeldayem, Abdul-Rahman M. A. Mohamed, Ali Sobhy, Mohamed A. El-Mokhtar, Magdy M. Afifi, Samah M. Fathy, Serageldeen Sultan

**Affiliations:** 1 Department of Microbiology, Faculty of Science, Al-Azhar University, 71524 Assiut, Egypt; 2 Department of Zoology, Faculty of Science, Al-Azhar University, 71524 Assiut, Egypt; 3 Department of Clinical Pathology, Faculty of Medicine, Al-Azhar University, 71524 Assiut, Egypt; 4 Department of Medical Microbiology and Immunology, Faculty of Medicine, Assiut University, Assiut, Egypt; 5 Gilbert and Rose-Marie Chagoury School of Medicine, Lebanese American University, Byblos Campus, Lebanon; 6 Department of Botany and Microbiology, Faculty of Science, Al-Azhar University, Nasr City 11884, Cairo, Egypt; 7 Department of Zoology, Faculty of Science, Fayoum University, Fayoum, Egypt; 8 Department of Microbiology, Virology Division, Faculty of Veterinary medicine, South Valley University, 83523 Qena, Egypt

**Keywords:** COVID-19, Clinical implications, Mutation, SARS-CoV-2, Spike Protein, Variants

## Abstract

The severe acute respiratory syndrome coronavirus-2 (SARS-CoV-2) infection is the cause of coronavirus disease 2019 (COVID-19); a severe respiratory distress that has emerged from the city of Wuhan, Hubei province, China during December 2019. COVID-19 is currently the major global health problem and the disease has now spread to most countries in the world. COVID-19 has profoundly impacted human health and activities worldwide. Genetic mutation is one of the essential characteristics of viruses. They do so to adapt to their host or to move to another one. Viral genetic mutations have a high potentiality to impact human health as these mutations grant viruses unique unpredicted characteristics. The difficulty in predicting viral genetic mutations is a significant obstacle in the field. Evidence indicates that SARS-CoV-2 has a variety of genetic mutations and genomic diversity with obvious clinical consequences and implications. In this review, we comprehensively summarized and discussed the currently available knowledge regarding SARS-CoV-2 outbreaks with a fundamental focus on the role of the viral proteins and their mutations in viral infection and COVID-19 progression. We also summarized the clinical implications of SARS-CoV-2 variants and how they affect the disease severity and hinder vaccine development. Finally, we provided a massive phylogenetic analysis of the spike gene of 214 SARS-CoV-2 isolates from different geographical regions all over the world and their associated clinical implications.

## Background

The first two decades of the 21st century have been challenged with several new viral outbreaks including avian influenza virus H5N1 (2006), swine influenza virus H1N1 (2009), severe acute respiratory syndrome coronavirus (SARS-CoV) in 2003, Middle East respiratory syndrome coronavirus (MERS-CoV) in 2012, Ebola virus in 2014, Zika virus (2016), and the most recently emerged coronavirus disease 2019 (COVID-19) in 2019. COVID-19 was declared a public health international emergency by the World Health Organization (WHO) on January 31, 2020. It has affected nearly 189 countries/territories with more than 761,071,826 confirmed cases, and around 6,879,677 human deaths and a total of 13,260,401,200 vaccine doses have been administered since March 20, 2023 (WHO, 2023) [1].

**Fig. 1:**
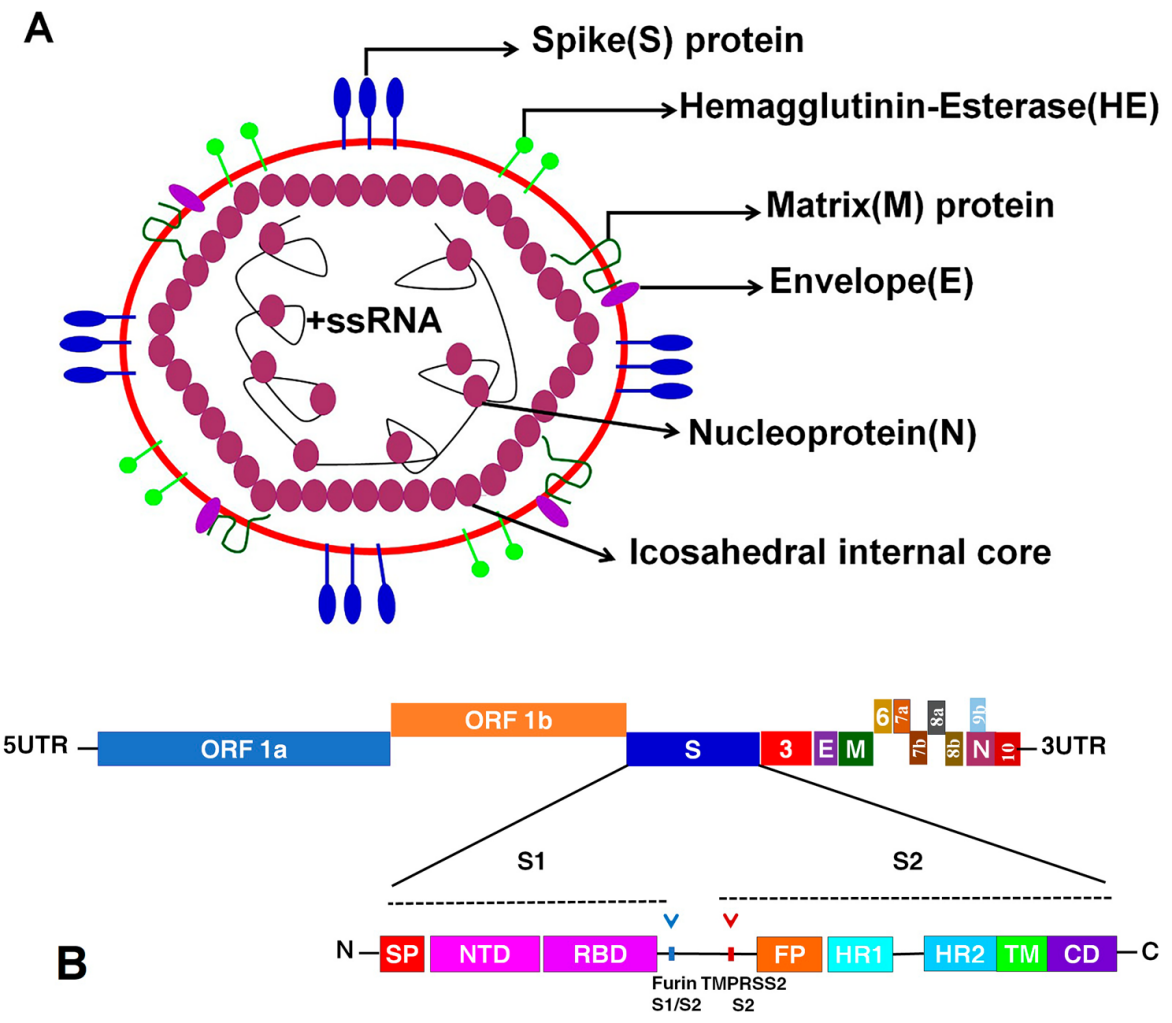
Schematic diagram showing the viral particle structure of SARS-CoV-2 **(A)**, and the genome organization of SARS-CoV-2 genes **(B)**. The structural components of the spike (S) protein; S2 contains signal peptide (SP), N-terminal domain (NTD), and receptor binding domain (RBD). The S1 contains fusion peptide (FP), heptad repeat domains (HR1 and HR2), trans-membrane domain (TMD) and cytoplasmic domain (CD). The arrowhead indicated the furin and TMPRSS2 cleavage sites. **(B)** The genome organization of SARS-CoV-2 genes

Coronaviruses (CoVs) are a family of enveloped, single-stranded, and positive-sense Ribonucleic acid (RNA) viruses (Fig. 1A). CoVs infect many mammals including humans and cause a wide variety of diseases including respiratory, enteric, hepatic, and neurological diseases [2]. They are zoonotic infections with animal origin and have the largest non-segmented RNA viral genome (∼ 30 kb). CoVs belong to the order *Nidovirales* in the family *Coronaviridae* and sub-family *Orthocoronavirinae* and include four genera: alpha-, beta-, gamma-, and delta-coronaviruses [3, 4].

Because of high genomic recombination, CoVs are rapidly evolving and affect a wide host range. It has been reported that all CoVs genera can infect animals [5]. Generally, *Alphacoronavirus* and *Betacoronavirus* infect mammals while *Gammacoronavirus*, and *Deltacoronavirus* infect both birds and mammals [6]. Interestingly, CoVs infection in human is only associated with *Alphacoronaviruses* and *Betacoronaviruses* genera [7]. CoVs that are able to infect human include: Human coronavirus NL63 (HCoV-NL63), Human coronavirus 229E (HCoV-229E), Human coronavirus OC43 (HCoV-OC43), Human coronavirus HKU1 (HCoV-HKU1), SARS-CoV, MERS-CoV, and SARS-CoV-2. HCoV-NL63 and HCoV-229E belong to *Alphacoronaviruses*, while HCoV-OC43, HCoV-HKU1, SARS-CoV, MERS-CoV, and SARS-CoV-2 belong to the *Betacoronavirus* [7, 8].

Human coronaviruses are commonly transmitted via the respiratory tract and most of them cause a mild infection like respiratory distress and diarrhea. Based on their pathogenicity, two alpha-CoVs (HCoV-229E and HCoV-NL63) and two beta-CoVs (HCoV-OC43 and HCoV-HKU1) cause a mild infection, while SARS-CoV, MERS-CoV, and SARS-CoV-2 are highly pathogenic to humans and cause severe infection in the lower respiratory system with the high potentiality of fatal respiratory diseases [7, 9–12].

Three Human CoVs devastating outbreaks have been recorded until now: the 2003 SARS-CoV pandemic, the 2012 MERS pandemic, and the 2019 SARS-CoV-2 pandemic [13–16]. SARS-CoV-2 infection was initially described in 2019 in Wuhan, China as severe respiratory distress with suspected animal origin. The intermediate host for SARS-CoV-2 is largely unknown. Recent reports indicate that SARS-CoV-2 has undergone several mutations [17–21]. These mutations greatly impact not only disease manifestations and outcomes, but also the performance of vaccines, therapeutic medications, diagnostic tools, and other public health managements. In this review, we aimed to survey and summarize the currently available information about the SARS-CoV-2 outbreak, viral proteins, and genomic mutations, and their functions during infection.

## COVID-19 in children

Compared to other age groups, COVID-19 is less common in children and infection is mainly asymptomatic. It has been reported that around 86% of COVID‐19 infected children in China remained undiagnosed which may constitute a risk for infection in elderly people [22]. Therefore, a small number of COVID-19 cases has been reported among children [23].

In a retrospective study of respiratory infections conducted in January 2020 (early in the epidemic) in central Wuhan showed that among 366 children, SARS-CoV-2 was detected in only 6 (1.6%) children [24]. The Chinese novel coronavirus pneumonia emergency response epidemiology team analyzed 72,314 subjects and reported that only 2% out of 44 672 COVID-19 patients were children (0‐19 years), of them 0.9% were under the age of 10 years [22]. In Italy only 1.2% of 22,512 Italian COVID‐19 cases were children and no deaths among the children or adults below the age of 30 years were reported [25]. In the United States, by March 2020, only 5% of 4226 cases were children. Generally, children constitute < 1% of hospitalized US patients [22].

Generally, the symptoms in children are less severe than in adults. A study from the Wuhan Children’s hospital reported cough, pharyngeal erythema, and a fever of at least 37.5 °C to be the most common symptoms [26]. Similarly, another study analyzed 2143 COVID-19 children’s cases and reported that the most common symptoms included fever, cough, sore throat, sneezing, myalgia, fatigue, and sometimes wheezing [27]. The other reported minor symptoms included diarrhea in 8.8% of the infected children, fatigue (7.6%), rhinorrhea (7.6%), and vomiting (6.4%) [26]. Another cohort study was performed including National Health Service (NHS) hospitals in England, Wales, and Scotland. It compared the rate of children and young people admission between the first and second wave of the disease in the UK. It showed that the severity of infection had not changed and that about 20% of the admitted children had asymptomatic/incidental SARS-CoV-2 infection [28]. In conclusion, the prevalence and the severity of COVID-19 are minor in children as compared to elderly people.

## Organs impairment in COVID-19

COVID-19 is a multi-organ destroying disease that damages the lungs as the primary organ and affects other organs such as the heart, kidney, and liver. Long-term deterioration to the alveoli in the lungs with consequent respiratory complications was attributed to lung inflammation resulting from COVID-19 infection. Direct and indirect cardiovascular disorders following COVID-19 infection were recorded, including myocardial injury, acute coronary syndrome, cardiac arrhythmias, cardiomyopathy, cardiogenic shock, and thromboembolic difficulties [29]. Moreover, the out-of-hospital cardiac arrest elevated approximately by 60% throughout COVID-19 pandemic in relation to the comparable time in 2019 [29]. Regarding the brain, Covid-19 can lead to strokes, seizures, and temporary paralysis and may also elevate the danger of Parkinson’s disease and Alzheimer’s disease [30].

Initially, a low incidence of acute kidney diseases was reported in COVID-19 patients whereas more recent reports indicate the opposite outcome. It was documented in a study of 59 patients with Covid-19 that 34% of patients developed extensive albuminuria on the first day of hospital stay and 63% of them revealed proteinuria during their hospitalization. Furthermore, inflammation and edema in the kidney as indicated by diminished renal density on CT scan have also been revealed in some patients. Lately, blood in urine, blood urea nitrogen, and upregulated serum creatinine were reported. Despite the mechanism of renal involvement is not elucidated, hypotheses were proposed including cytokine storm or direct cell destruction by SARS-CoV-2 infection. Another outcome that suggests renal involvement in COVID-19 is the virus appearance in the urine samples of many infected patients [31].

Abnormalities of liver functions have been reported in COVID-19 infected patients [32]. The levels of Alanine aminotransferase (ALT), Aspartate transaminase (AST), bilirubin, and Lactate dehydrogenase (LDH) were significantly elevated in severe cases compared with milder cases [33–38]. However, autopsy examination showed that the COVID-19 patient did not report serious macroscopic changes in the liver appearance [39]. In another study, examination of a liver biopsy from dead COVID-19 patients revealed moderate micro-vesicular steatosis, and mild portal and lobular activities [40]. Zhang et al. [41] have shown mild sinusoidal dilatation and a low level of infiltrating lymphocytes in the liver tissues. In addition to that the expression of angiotensin converting enzme 2 (ACE2) receptors in the liver cells is very weak and SARS-CoV-2 infection of the hepatocytes affects liver functions insignificantly [42]. However, the changes that occurred in the liver tissues of COVID-19 patients could be the result of other pathological causes such as hypoxemia, thrombi formation, inflammatory mediators’ secretion, or drug-induced liver injuries [43–46]. Given the fact that severe COVID-19 cases are associated with hypoxemia, liver tissues may also be affected accordingly leading to abnormalities of liver functions. Another explanation for the damage to the liver is the administration of nonsteroidal anti-inflammatory drugs as analgesics by patients before hospital administration [47, 48].

## Comorbidities associated with COVID-19 severity

Although limited data are available about COVID-19, it was documented that comorbidities propagate the probability of infection [49]. People at high risk of severe infection include old age with chronic diseases as well as patients with uncontrolled medical conditions such as diabetes mellitus; hypertension; cancer; liver, kidney, lung disorders; smokers; people receiving grafts; and patients under chronic steroids’ treatments. A meta-analysis investigation was performed on COVID-19 comorbidities with a total of 1786 patients [50]. Hypertension was the most prevalent comorbidity (15.8%). Cardiovascular and cerebrovascular disorders (11.7%) and diabetes (9.4%) are also the most common comorbidities [50, 51]. Meanwhile, the same study reported that coexisting infections with HIV and hepatitis B (1.5%), malignancy (1.5%), respiratory illnesses (1.4%), renal disorders (0.8%), and immunodeficiencies (0.01%) were the less common comorbidities. A study in Australia including 1625 patients diagnosed with SARS-CoV-2 infection was performed. It has been documented that there was a significant correlation between comorbidities, including chronic respiratory disease, chronic cardiac disease, and morbid obesity with the disease severity and intensive care unit (ICU) admission [52]. Another cohort study of 7337 COVID-19 individuals with and without type 2 diabetes demonstrated that those with type 2 diabetes necessitated intensive interventions during their hospital stay compared with nondiabetic patients [53]. The previous study concluded that there was a general declined multiple adverse effects and death for patients with blood glucose, particularly for those in the range of 3.9 to 10.0 mmol/L [53]. Reduced blood glucose control was ascribed with significantly higher risk of complications and mortality [53]. Chronic obstructive pulmonary disease (COPD) is another comorbidity that has been related to the severity of the disease. A meta-analysis of multiple studies in China reported that patients suffering from COPD who were diagnosed with COVID-19 revealed a four-fold increase in death [54]. The same study found no significant correlation between smoking and COVID-19 severity outcomes [53]. A prothrombotic coagulopathy may be the cause of patients’ infection with SARS-CoV-2 who suffers from respiratory failure and acute respiratory distress syndrome (ARDS) [55]. Dispersed microthrombi in the pulmonary vasculature were discovered during the autopsy of COVID-19-related deaths, indicating an occlusive reason of respiratory failure [55]. It has been noted that 38 to 100% progress in three cases with COVID-19-related ARDS and respiratory failure, after intravenous (IV) antithrombotic alteplase treatment [55]. However, the outcomes were only temporary in two out of the three cases, as recovery failed after treatment [55]. Subsequently, further research on the use of anti-thrombolytic therapy is required. Different study was done on 342 hospitalized patients with COVID-19. It has been revealed that no relation was detected between proteinuria, which is also an indicator of chronic kidney disease (CKD), and COVID-19 severity [56].

A cohort study of 2007 COVID-19 cases reported that cancer patients had a greater risk of severe complications than those without cancer (39% vs. 8%, *p* = 0·0003) [57]. The American Association for Cancer Research delivered a report proving the effect of COVID-19 on cancer patients [58]. Although the case fatality rate in the case of COVID19 is relatively low in the general population, it could be doubled among cancer patients [59]. In a retrospective case study on 28 COVID19-infected cancer patients, 53.6% of the patients suffered from severe manifestations and the mortality rate reached 28.6%. Marked deterioration of the patient clinical courses occurred when the antitumor treatment was administered [60]. In conclusion, cancer patients with COVID-19 infection, particularly those with lung cancer, show deteriorating conditions and poor outcomes. Blanco-Melo, et al. [24] reported in a retrospective case study involving 138 patients that 41.3% of the patients have acquired the COVID-19 infection during hospitalization. They found that 5 patients of this cohort were from the oncology department. It is recommended that cancer patients undergoing antitumor care should be actively tested for COVID-19 infection and should not be allowed to take immunosuppressive therapies or at least decrease their dosages in cases of infection with SARS-CoV-2 [60].

## SARS-CoV-2 genomic structure and proteins

### Genomic structure

SARS-CoV-2 belongs to *Sarbecovirus* in the genus *Betacoronavirus* and has a 26 to 32 kb length positive single-stranded RNA genome encoding 9860 amino acids (aa). The SARS-CoV-2 genome contains two untranslated regions (UTRs); 265 nucleotides at 5′ end and 358 nucleotides at 3′ end, and 6 to 11 open reading frames (ORFs) including ORF1a/b, Spike(S), Envelope(E), Membrane or Matrix (M), Nucleocapsid (N), ORF3b and ORF8 [24, 51] (Fig. 1A and B). There are several stem-loop structures in the SARS-CoV-2 genome that are critical for replication and transcription of the viral genome. The ORF1a/b at the 5′ end is the longest among SARS-CoV-2 ORFs comprises a two-thirds of the virus genome and encode two polyproteins, ORF1a and ORF1ab, that are further processed into 16 non-structural proteins (NSP)1–16 [24, 61]. The S, E, M, and N proteins located at the 3-teminus of the SARS-CoV-2 genome are the major viral structural proteins (SPs) [62, 63]. The ORF1a and ORF1a/b proteins are critical for viral transcription and replication, whereas S, E, and M proteins mediate cellular entry, morphogenesis, assembly, and budding of the virus particles, respectively.

### SARS-CoV-2 proteins

#### SARS-CoV-2 SPs

##### Spike (S) protein

The S protein of SARS-CoV-2 is a glycoprotein located at the surface of viral particle (Fig. 1A). It is the viral fusion protein that mediates viral attachment and internalization to the host cells via binding to ACE2 receptors expressed on the surface of the host cell [64–66]. The S protein is a type I membrane glycoprotein and has a crown shape which gives coronaviruses their major morphological characteristics. It is the major determinant of antiviral immune repose and critical for developing viral-neutralizing antibodies against coronaviruses [67]. It is 1300 aa in length and 150 KDa in size and has three major domains: an extracellular domain, a trans-membrane domain (TMD), and an intracellular domain. It is composed of two subunits, a receptor-binding subunit (S1) which binds to the receptor on the host cell surface and mediates viral attachment, and a membrane-fusion subunit (S2) which fuses the host and viral membranes, allowing viral entry into host cells [68, 69] (Fig. 1B). The SARS-CoV-2 S2 subunit is highly conserved with 90% sequence identity to human SARS-CoV and bat SARS-like CoVs, while the S1 subunit is less conserved with 70% sequence identity to other SARS-CoVs [62, 68]. The receptor-binding domain (RBD) of the subunit S1 directly interacts with the peptidase domain of ACE2 receptors. Therefore, RBD is the critical determinant of viral host range, tropism, and infectivity [64, 69].

##### The membrane (M) protein

SRAS-CoV-2 M glycoprotein is a type III trans-membrane glycoprotein and the most abundant protein on the surface of viral particles. It has 39.2%, 90.1%, 98.2%, and 98.6% homology to that of MERS, SARS-CoV-1, pangolin SARS-CoV, and bat SARS-CoV, respectively [22, 70, 71]. The M protein of SARS-CoV-2 is 25 to 35 KDa in molecular weight and 230 aa in length. It is composed of three major domains; a long C-terminus domain that is imbedded inside the envelope, a triple trans-membrane spanning domain, and a short N-terminus domain protruding outside the viral particle [67, 70, 71] (Fig. 1A).

The M protein is essential for the formation and assembly of the virus particles. The Mutated M protein has been shown to lack the ability to form virus-like particles [67, 72]. Throughout interacting with other viral SPs such as S, E, and N proteins, the M protein mediates a variety of critical functions during coronavirus infection including proliferation, replication, and immune evasion [70]. The binding of the M protein to the N protein has been shown to stabilize the N protein-RNA complex and promote viral assembly [73, 74]. Using *in silico* analysis has revealed that the M protein of SARS-CoV-2 has a Semi- sugars will eventually be exported transporter (SWEET) sugar transporter-like structure and could influence the glycosylation of another viral glycoprotein like S glycoprotein [71]. Based on this result, it can be assumed that M protein could play a role during virus attachment and entry into the host cell [71].

##### The nucleocapsid (N) protein

The N protein has a molecular weight of 43–50 KDa with helical capsid symmetry that binds to genomic viral RNA to form helical ribonucleoproteins. It is composed of highly conserved three distinct domains; the N-terminal domain (NTD, residues 45–181) and the C-terminal domain (CTD, residues 248–365) that are linked by an intrinsically central disordered domain (a serine/arginine-rich domain). The N protein binds viral RNA by its NTD and the CTD which are rich in positive aa [75–77].

The N protein has been shown to play a critical role in the coronavirus life cycle throughout involving multiple functions including, replication, transcription, and packaging of the viral RNA genome [78–80]. Serological analysis of sera from SARS-infected patients revealed a high level of N protein-specific IgG antibodies which indicated the high immunogenicity of this protein [79, 81]. Interestingly, antibodies against the SARS-CoV N protein are more effective than antibodies generated against other SPs because of their higher sensitivity and longer persistence [82, 83]. These results demonstrated that N protein can be used as a target to develop an effective vaccine against SARS-CoV-2 [80, 84, 85].

##### The envelope (E) protein

The SARS-CoV E protein is a short less abundant viral membrane protein and the smallest structural protein in the viral particle with 74–109 aa length and molecular weight 8.4–10.9 KDa [86–89]. It consists of three domains: negatively charged hydrophilic NTD, uncharged hydrophobic TMD, and variably charged hydrophilic CTD [86, 87]. The CTD of E protein has a post-synaptic density protein-95/Discs Large/Zonula occludens-1 (PDZ)-binding motif (PBM) which binds to Protein Associated with Caenorhabditis elegans Lin-7 protein 1 (PALS1) [90]. PALS1 is a tight junction-associated protein that belongs to PDZ domain-containing proteins that work as scaffolds for signaling proteins [86, 88, 90, 91].

Consequently, the E protein has been shown to be necessary for the production and maturation of virus particles through interaction with other viral proteins [86, 87]. The interaction of the E protein with the viral M protein is important for viral assembly [87, 92, 93]. Similarly, the TMD of E protein has been proven as a crucial motif for virus release and must be expressed along with viral N, and M proteins for efficient assembly and release of virus-like particles [93–95]. Moreover, SARS-CoV E protein is a determinant of viral pathogenesis throughout its role in the elevation of viral virulence and exacerbation of the antiviral immune response [96, 97]. Pending of E protein PDZ-binding motif to cellular protein syntenin leads to redistribution of syntenin from the nucleus to cellular cytoplasm and activation of p38 MAPK pathway which leads to the overexpression of inflammatory cytokines and exacerbation of the virus infection [96]. Interestingly, viral-associated immunopathology was significantly alleviated in cells infected with SARS-CoVs that is lacking E protein PBM and in cells where syntenin has been silencing by using syntenin-specific siRNAs [86, 96].

#### NSPs

The SARS-CoV-2 genome encodes for NSP1-NSP16 that regulate viral transcription and replication. These NSPs are encoded by ORF 1a/b which is located at the 5′ end of the viral genome (Fig. 1B). The ORF 1a/b initially translated into two primary polyproteins, ORF1a and ORF1ab, that are sequentially processed to 16 NSPs throughout autoproteolytic cleavage [61, 68]. NSP1–NSP10 and NSP12–NSP16 are products of ORF 1a/b while NSP11 is processed from the cleavage of ORF 1a [98, 99]. Coronaviruses NSPs are indispensable for replication and transcription of the viral RNA genome as shown in Table 1.

**Table 1:** Functions of SARS-CoV-2 Non-structural proteins

Protein	Functions during viral infection
NSP1	Promote translation inhibition and cellular mRNA degradation [142, 143]. Block mRNA entry channel on the 40 S ribosome [142, 143]. Prevent physiological conformation of the 48 S preinitiation complex [142]. Inhibit antiviral immune response [144–146]. Mediate immune evasion [144, 146].
NSP2	Potential role in viral pathogenesis by increasing viral ability of contagious [59]. Has a suggested role in calcium homeostasis and mitochondria biogenesis [147]. Vesicle trafficking [148].
NSP3	Part of replicase–transcriptase complex in the infected cell [148] Contains papain-like protease (SCoV2-PLpro) activity that cleaves Nsp1, Nsp2 and Nsp3 from the viral polypeptide and regulates the virus-induced cytopathogenic effect and antiviral innate immunity [149–151]. Essential for replication and transcription of the viral genome [149, 152]. Nsp3 Mac1 domain binds to ADP-ribose (ADPr) and catalyzes the hydrolysis of ADPr-1′′ phosphate which is linked to SARS-CoV-2 associated cytokine storm and viral evasion of the innate immune response [153–155].
NSP4	Potential role related to membrane rearrangement and formation of the double-membrane vesicles (DMVs) and viral replication [156, 157]. Has a suggested role in calcium homeostasis and mitochondria biogenesis [147].
NSP5	3 C-like protease proteolytically cleaves viral polyprotein [148, 158]. Acts as epigenetic and gene-expression regulators [148].
NSP6	Suppress IFN-I signaling [145]. Formation of autophagosome in the infected cell [159–161]. Vesicle trafficking [148]. Expected to involve in membrane rearrangement and formation of DMVs [159]. Interacts with the sigma receptor that has been linked to lipid remodeling and the stress response of the endoplasmic reticulum (ER) [148, 159].
NSP7	Essential cofactor with NSP8 for NSP12 which are RNA polymerase [162–165]. Vesicle trafficking [148].
NSP8	Essential cofactor with NSP7 for NSP12 which are RNA polymerase [162–165]. Involve in the regulation of lipid modification, RNA processing, epigenetic and gene expression [148].
NSP9	RNA binding protein [166].
NSP10	Form 2’-O-methyltransferase protein complex with NSP16 which catalyzes the methylation of viral RNA cap at the ribose 2′-O position [167, 168]. Vesicle trafficking [148].
NSP11	Intrinsically disordered proteins contribute to the host cytosolic membrane affinity/interaction [169].
NSP12	RNA-dependent RNA polymerase (RdRp) [162–165].
NSP13	Helicase, 5′ triphosphatase that unwinds RNA helix and hydrolyzes NTPs [170–173]. Repress interferon production and signaling [174]. Acts as epigenetic and gene-expression regulators [148].
NSP14	3’-5’ exoribonuclease that is critical for proofreading and synthesis of viral RNA [175]. N-7 methyltransferase that involves in the capping of viral RNAs [175]. Repress interferon production and signaling [174].
NSP15	Nidoviral RNA uridylate-specific endoribonuclease cleavage of RNA at the 3’-ends of uridylates to limit the accumulation of viral RNA and inhibit cellular sensing of the viral genome [176–178]. Vesicle trafficking [148]. Repress interferon production and signaling [174].
NSP16	Form 2’-O-methyltransferase protein complex with NSP10 which catalyzes the methylation of viral RNA cap at the ribose 2′-O position [167, 168].

## SARS-CoV-2 genetic diversity and their clinical implications

Sequencing of the whole genome of SARS-CoV-2 revealed that the virus has 96.2% similarity to that of a bat SARS-related coronavirus (SARSr-CoV; RaTG13) collected in Yunnan province, China, but has low similarity to that of SARS-CoV-1 (∼ 79%) and MERS-CoV (∼ 50%) [68, 100]. Although, antigenic drift has been frequently reported in human coronaviruses such as HCoV-229E [101], HCoV-OC43 [102], and SARS-CoV-1 [103, 104], to date there is no antigenic drift reported on SARS-CoV-2 [105]. However, emerging evidence indicated that antigenic or vaccine escape SARS-CoV-2 mutants with high immunological resistance are likely to appear [105, 106]. The emergence of viral antigenic mutants will greatly affect the development of vaccines and immunotherapeutic agents.

### SARS-CoV-2 lineages and clades

Based on genetic analysis of the publicly available SARS-CoV-2 genome sequences, there are two major lineages of SARS-CoV-2 designated as linage A and B [107], or S and L, respectively [108]. The lineage B is the most prevalent (∼ 70%), while the lineage A is less dominant (∼ 30%) and has a high correlation to animal coronaviruses [107, 108]. Based on single nucleotide polymorphisms (SNPs) analysis there is a higher mutation rate in the L lineage of SARs-CoV-2 viruses than S lineage [108].

**Fig. 2:**
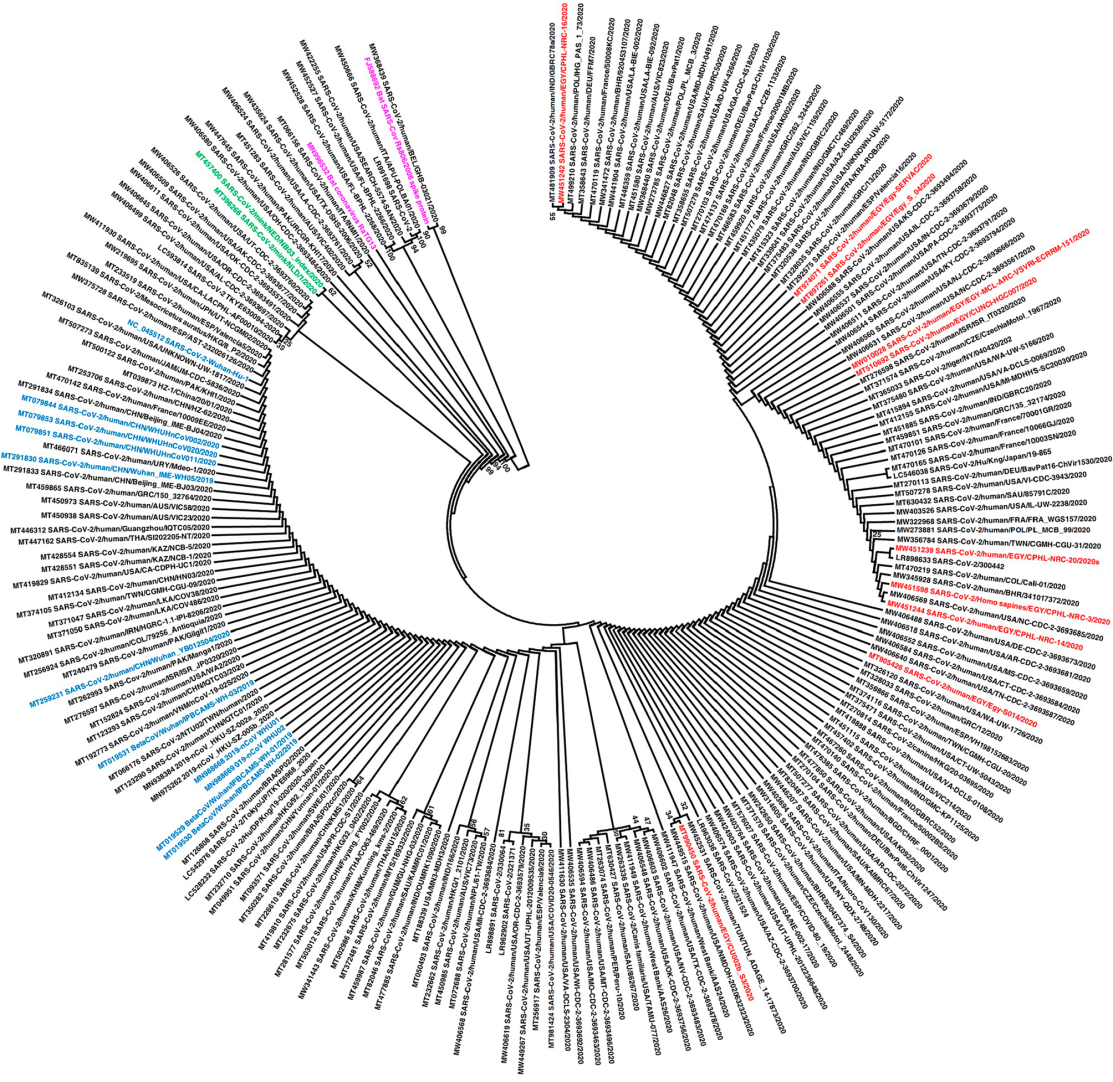
The phylogenetic analysis of the full spike (S) protein genes coding region of 214 SARS-CoV-2 sequences deposited in GenBank from various countries all over the world. The tree was constructed using the maximum-likelihood method in the MEGA6 software. The evolutionary distances were computed by General Time Reversible model and bootstrap 1,000 replicates with complete deletion of the gap and missing data. The SARS-CoV-2 sequences from Wuhan, China (blue), bat RaTG13 (pink), mink (green) and Egypt (red). The accession numbers, source of sequence, date and country of origin are shown in the sequence labels

The lineage or clade is a group of viruses that come from one ancestor and are genetically similar. Also, the viruses that showed different specific mutations were assigned as lineage or clade. Figure 2 shows two major lineages from which various virus groups (sublineages) that are similar (not identical) to each other are formed.

Based on the geographical occurrence (Fig. 2), the frequencies of the predominant mutations and genome sequence identities, SARS-CoV-2 lineages A and B were proposed to be classified into several sub-lineages including sub-lineages A.1 (A.1.1 and A.1.3), A.2, A.3, A.4, A.5, A.6, B.1 (B.1.1, B.1.3, B.1.5, B.1.6, B.1.7, B.1.12, B.1.19, B.1.22, B.1.23, B.1.26, B.1.29, B.1.30 B.1.31, B.1.32, B.1.33, B.1.34 B.1.35, B.1.36, B.1.37, B.1.38, B.1.39, B.1.40, B.1.41, B.1.43 B.1.66, B.1.67, B.1.69, B.1.70, B.1.71), B.2 (B.2.6, B.2.2, B.2.7, B.2.4, B.2.5, B.2.1), B.3, B.4, B.5, B.6. B.7, B.9, B.10, B.13, B.14, B.15, and B.16 [109]. Similarly, there are five designated clades of SARS-CoV-2: O, V, G, GR, and GH clade. The clade G and its mutated GR and GH are predominantly circulated and occupied ∼ 74% of the obtained genome sequences [110–112].

### SARS-CoV-2 genome mutations

Several mutations have been reported in the SARS-CoV-2 genome [113–115]. The viral spike protein G614 mutant is the most common and pathogenic mutation of SARS-CoV-2 [116]. Most of the G614 SARS-CoV-2 mutants belong to the G clade and patients infected with this mutant show higher virus load and low cycle threshold (Ct) value than those infected with SARS-CoV-2 D614 mutant but the severity of the disease did not change in comparison with SARS-CoV-2 viruses bearing a D614 mutation [117]. The D614G variant frequently occurs in sub-lineages B.1, B.1.1, B.1.1.1, B.1.1.10, B.1.5, and B.1.5.4. While lineages B, B.2, B.2.1, and B.2.5 have G251V substitution in the NSP3, the sub-lineages A.1, A.2, and A.5 are associated with L84S mutation in the ORF8 [118]. Nine lineages have been identified in Malaysians including B.6, B, B.1.1, B.1, A, B.1.1.1, B.2, B.1.36, and B.3 according to their incidence in 115 SARS-CoV-2 sequences. Among these lineages, only B.1, B.1.1, B.1.1.1, and B.1.36 lineages have the D614G mutation in the S protein that may increase the SARS-CoV-2 infectivity [119].

Similarly, three deletions have been reported in the genomes of SARS-CoV-2 from Japan (Aichi), the USA (Wisconsin), and Australia (Victoria) [120]. Two of these deletions (3 nt and 24 nt) were in the ORF1ab polyprotein, and one deletion (10 nt) was in the 3′ end of the genome. In addition, there are 93 nucleotide substitutions induced 42 aa mutations in the entire genome including the ORF1ab polyprotein (29 aa), the N protein (8 aa), the M protein (1 aa), and the S (4 aa) surface glycoprotein. It is worth noting that D354, Y364, and F367 aa mutations located in the SARS-CoV-2 S surface glycoprotein RBD may affect the virus antigenicity. aa analysis revealed that the six critical aa (L455, F486, Q493, S494, N501, and Y505) in the RBD of the S protein were completely conserved between SARS-CoV-2 and GD Pangolin-CoV. While only one aa residue was conserved among SARS-CoV-2, SARS-CoV (Y505), and SARSr-CoV; RaTG13 (L455) [108]. Thus SARS-CoV-2 exhibited a higher binding affinity to ACE2 receptors than SARS-CoV [69]. The aa motifs such as G493 and N501 show favorable interaction and compatibility with human ACE2 receptors [65]. Moreover, SARS-CoV-2 has more transmissibility than SARS-CoV which leads to a speedy increase in the confirmed cases worldwide [11]. Two groups of aa mutations in SARS-CoV-2 RBD were identified: the first group possesses F342L and R408I, and the second group has N354D, D364Y, V367F, and W436R aa mutations. The frequency of F342L in RBD among 6 isolates indicated their evolving as a novel sub-lineage and supposed worthy for virus transmission.

In addition to the S protein, mutations in other viral SPs have been shown to modulate virus infection and disease progression [121]. R203K, G204R, R203M, and T205I mutations in viral N protein increased virus infectivity, and disease severity [122, 123]. Similarly, mutations in E (e.g., T9I) and M (e.g., I82T) proteins have been emerged recently. These mutations to impact virus thermodynamic properties and modulate virus infection [124–126].

### SARS-CoV-2 variants

Multiple variants of SARS-CoV-2 are continually reported worldwide including variants from the United Kingdom, South Africa, and Brazil [127, 128]. Most of these variants have greatly altered transmission, virulence, infection outcome, and infection control strategies, especially diagnostic tests, and vaccines development as shown in Table 2.

**Table 2:** Clinical implications of SARS-CoV-2 variants and mutations

Variant/Mutation	Clinical implications
The Spike protein D614G mutation	Increase the infectivity of SARS-CoV-2 by reducing viral neutralization by adaptive immune response [115, 116, 179].
20I/501Y.V1/VOC 202,012/01 variant	Higher transmissibility rate and increased risk of death [180].
20 H/501Y.V2 variant	Higher transmissibility rate and more severe disease [127, 128]
20 J/501Y.V3/ P.1 variant	Potential higher transmissibility and re-infection rates [127, 136, 181]

WHO’s virus evolution working group (VEWG) classified SARS-CoV-2 into two major variants: a variant of concern (VOC) and a variant of interest (VOI). VOC is characterized by increased transmission, changed clinical manifestations, and decreased efficiency to available vaccines, therapeutics, and public health measurements. The VOI has been reported in many countries to be responsible for community transmission of COVID-19 [129–131]. Each of the Global Initiative on Sharing All Influenza Data (GISAID), NEXTstrain, Pango, and WHO have designated their unique nomenclature systems for naming and tracking the VOC and VOI as shown in Table 3. To avoid community or country variants confusion, WHO has recommended using of Greek letters (Alpha, Beta, Gamma…etc.) [131].

**Table 3:** Nomenclature of SARS-CoV-2 variants

Nomenclature					Origin	Date	Major variant
WHO	**Pango**		**GISIAD**	**Nextstrain**			
Alpha	B.1.1.7		GR/501Y.V1(GRY)	20I/S:501Y.V1	UK	18/12/2020	VOC
						9/3/2022	
Beta	B.1.351 B.1.351.2 B.1.351.3		GH/501Y.V2	20 H/S:501Y.V2	South Africa	18/12/2020	
						9/3/2022	
Gamma	P.1 P.1.1 P.1.2 P.1.4 P.1.6 P.1.7		GR/501Y.V3	20 J/S:501Y.V3	Brazil	11/1/2021	
						9/3/2022	
Delta	B.1.617.2 AY.1 AY.2 AY.3 AY.3.1		G/452R.V3	21 A/S:478 K	India	11/5/2021	
						7/6/2022	
Omicron parent lineage		B.1.1.529	GR/484A	21 K	Multiple countries	26/11/21	
						14/3/2023	
Epsilon	B.1.427/B.1.429		GH/452R.V1	20 C/S:452R	USA	5/3/2021	VOI
						6/7/2021	
Zeta	P.2		GR	20B/S:484 K	Brazil	17/3/2021	
						6/7/2021	
Eta	B.1.525		G/484K.V3	21D/S:484 K	Several countries	17/3/2021	
						20/9/2021	
Theta	P.3		GR	20B/S:265 C	Philippines	24/3/2021	
						6/7/2021	
Iota	B.1.526		GH/452R.V1	21 F/S:484 K	USA	24/3/2021	
						20/9/2021	
Kappa	B.1.617.1		G/452R.V3	21B/S:154 K	India	4/4/2021	
						20/9/2021	
Lambda	C.37		GR/452Q.V1	21G	Peru	14/6/2021	
Mu	B.1.621		GH	21 H	Colombia	30/8/2021	
						9/3/2022	

#### The B.1.1.7 lineage (alpha variant)

The VOC 2020/12/01 (20I/501Y.V1) variant also known as alpha or GR/501Y.V1 is a highly transmissible SARS-CoV-2 mutated virus and belongs to lineage B.1.1.7 [132]. This variant was first detected in the United Kingdom in Sep. 2020, and subsequently detected in the United States and Canada [132, 133]. This variant has an unusually large number of mutations (e.g., N501Y, A570D, and D614G in RBD, 69–70, and 144 deletions in S1, P681H near S1/S2 cleavage site, T716I, S982A, and D1118H in S2 (ECDC, 2020; CDC, 2021).

#### The B.1.351 lineage (Beta variant)

Beta Variant (also known as 20 H/501Y.V2, or GH/501Y.V2 variant) has been detected in South Africa in Oct. 2020 and belongs to the lineage B.1.351 [128]. Although it has some mutations (K417T, E484K, and N501Y in S1 protein RBD) like 20I/501Y.V1, it emerged independently from the 20I/501Y.V1 variant (CDC, 2021).

#### P.1 variant (Gamma variant)

The Brazilian P.1 variant (also known as 20 J/501Y.V3, Gamma, or GR/501Y.V3 variant) was detected in 42% of the sequenced samples in the Amazon region of Brazil in which 75% of the population was SARS-CoV-2 infected in Oct. 2020 [134]. The Gamma variant also has been detected in a traveler returning from Brazil to Italy, and in 4 Brazilian travelers in Tokyo, Japan as well as in the UK [127, 135, 136]. It has 17 unique mutations with 3 (K417T, E484K, and N501Y) of them in the RBD of S protein and 3 deletions and was clustered in B.1.1.28 lineage [137] (CDC, 2021). In addition, two SARS-CoV-2 sequences were identified in Nigeria belonging to the B.1.1.207 lineage and sharing only P681H mutation with the B.1.1.7 lineage coronaviruses (CDC, 2021).

#### The B.1.617.2 (Delta variant)

The B.1.617 lineage, also known as G/452.V3 or 21 A/S:478 K identified independently in Maharashtra, India where WHO designated B.1.617 and its sub-lineages, B.1.617.1 (Kappa), B.1.617.2 (Delta), and B.1.617.3 as VOC. The B.1.617 has seven mutations in the spike protein including D111D, G142D, L452R, E484Q, D614G, P614R, and P681R [138]. The double B.1.617 mutations in the spike protein RBD, E484Q, and L452R, are responsible for increasing transmission and infectivity, immune response evasion, and high affinity to the human (h)ACE2 receptors [139, 140].

#### The B.1.1.529 (omicron variant)

The B.1.1.529 lineage was also recognized as GR/484A or 21 K. The Omicron variant was the most prevalent lineage during 2022. It possesses five sub-lineages with BA.1 being the initial subtype that appeared in South Africa during winter 2021–2022 [4].

#### The B.1.427/B.1.429 lineage (Epsilon variant)

The B.1.427 and B.1.429 lineages (or GH/452R.V1, or 20 C/S:452R, or Epsilon variant) originated in California. They are characterized by S13I and W152C mutations in the NTD and the L452R mutation in the RBD of spike protein [141].

## Conclusions and recommendations

SARS-CoV-2 outbreak is still a major global public health problem. Finding novel therapeutic targets is necessary to combat the SARS-CoV-2 outbreak and its associated pathogenesis. New evidence indicated that viral proteins and their mutations are promising targets to inhibit SARS-CoV-2 infection as they are the main determinants of infection severity and outcome. A better understanding of the role of SARS-CoV-2 proteins and their mutations in COVID-19 progression is mandatory not only to uncover the mystery behind the variations in the outcomes of SARS-CoV-2 infection but also to design an effective vaccine and treatment regimens for SARS-CoV-2 infections and COVID-19 respectively. SARS-CoV-2 variants with public health concerns are continually reported worldwide. The emergence of these variants not only affects viral transmission and virulence, but also hinders vaccine development and production. Tracking and investigating the emerging SARS-CoV-2 variants are valuable tools to tackle the risk of SARS-CoV-2 transmission and to develop an effective vaccine. Future studies are required to investigate and understand the mechanisms behind the emergence of SARS-CoV-2 mutations and variants. Such studies would result in a novel approach toward overcoming the SARS-CoV-2 infection and pandemic.

## Data Availability

Not applicable.

## Abbreviations

COVID-19: Coronavirus disease-2019
SARS-CoV-2: Severe acute respiratory syndrome coronavirus-2
MERS-CoV: Middle East respiratory syndrome coronavirus
CoVs: Coronaviruses
HCoV-NL63: Human coronavirus NL63,HCoV-229E,Human coronavirus 229E
HCoV-OC43: Human coronavirus OC43
HCoV-HKU1: Human coronavirus HKU1
ACE2: Angiotensin-converting enzyme 2
ARDS: Acute respiratory distress syndrome
ALT: Alanine aminotransferase
AST: Aspartate transaminase
LDH: Lactate dehydrogenase
NSP: Non-structural proteins
UTRs: Untranslated regions
VOC: A variant of concern
VOI: A variant of interest
